# Supplementary material for: Preparation and Optical Study of 1-Formamido-5-Isocyanonaphthalene, the Hydrolysis Product of the Potent Antifungal 1,5-Diisocyanonaphthalene
Source: Int J Mol Sci. 2023 Apr 24;24(9):7780. doi: 10.3390/ijms24097780 (PMC10177923; doi:10.3390/ijms24097780)
Supplement: Supplementary file 1 [file ijms-24-07780-s001.zip › ijms-2340896-supplementary.pdf]

Supplementary Information

for

Preparation and Optical Study of 1-Formamido-5-Isocyanonaphthalene, the Hydrolysis Product of the Potent Antifungal 1,5-Diisocyanonaphthalene

Erika Kopcsik [1], Zoltán Mucsi [2,3,*], Bence Kontra [3,4], László Vanyorek [1], Csaba Váradi [2], Béla Viskolcz [1,2] and Miklós Nagy [1,*]

1 Institute of Chemistry, University of Miskolc, Miskolc-Egyetemváros, 3515 Miskolc, Hungary; ria.toth1@gmail.com (E.K.); kemvanyi@uni-miskolc.hu (L.V.); bela.viskolcz@uni-miskolc.hu (B.V.)

2 Advanced Materials and Intelligent Technologies Higher Education and Industrial Cooperation Centre, University of Miskolc, Miskolc-Egyetemváros, 3515 Miskolc, Hungary; kemcsv@uni-miskolc.hu

3 Department of Chemistry, Brain Vision Center, Liliom utca 43-45, 1094 Budapest, Hungary; bence.kontra@femtonics.eu

4 Department of Organic Chemistry, Semmelweis University, Hőgyes Endre utca 7, 1092 Budapest, Hungary

* Correspondence: zoltanmucsi@gmail.com (Z.M.); nagy.miklos@uni-miskolc.hu (M.N.)

Experimental

Reagents and solvents were purchased from Sigma Aldrich in reagent grade and used as received. Deuterated solvents were purchased from Eurisotop. The reactions were monitored by a Shimadzu LC-40D XR UPLC-MS system equipped with a SIL-40C XR autosampler, SPD-M40 photo diode array detector and an LCMS-2020 DUIS Mass Spectrometer operated in negative and positive ionisation modes. The separation was carried out on Ascentis® Express C18, 2 µm UHPLC column (L × I.D. 5 cm × 2.1 mm), at 40°C provided by a CTO-40s column oven. Gradient elution was used with either eluent 0.1% TFA in water (A) and 0.1% TFA in MeCN (B); or 0.4 g NH_4HCO_3 in 1 L water (A) and MeCN (B). For preparative HPLC an Armen SPOT Prep II instrument using UV detector (200-600 nm scan) equipped with a Phenomenex Gemini C18, 250×50.00 mm; 10 µm, 110A column was used. Gradient elution was employed using 0.4 g NH_4HCO_3 in 1 L water (A) and acetonitrile (B) as eluent system. The NMR spectra were recorded at 25 °C, in the solvent indicated, either on a Varian Mercury Plus spectrometer (Agilent Technologies, Santa Clara, CA, USA) at a frequency of 400 MHz (^{1}H) or 101 MHz (^{13}C), or on a Varian Unity INOVA spectrometer operating at a frequency of 500 MHz (^{1}H) or 126 MHz (^{13}C). Notations for the ^{1}H NMR spectral splitting patterns include singlet (s), doublet (d), triplet (t), broad (br) and multiplet/overlapping peaks (m). Chemical shifts of the resonances are given as δ values in ppm and coupling constants (J) are expressed in Hertz. HRMS spectra were recorded Xevo G2-XS QTof Mass Spectrometer, with the default ESI ionization method.

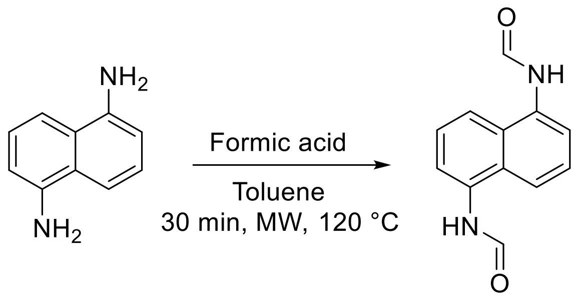

1 **2**

Synthesis of N,N'-(naphthalene-1,5-diyl)diformamide (2)[A]:

The mixture of naphthalene-1,5-diamine **1** (1.0 g, 6.3 mmol, 1 equivalent), toluene (10 ml) and 98% formic acid (1 ml, 25.3 mmol, 4 equivalent) was stirred in a microwave reactor at 120 °C for 30 minutes. After cooling down to room temperature, to the mixture with white crystals 10 ml dichloromethane was added and the volatile components were removed under reduced pressure. The residue was treated with a portion of toluene (10 ml) and evaporated again to give N,N'-(naphthalene-1,5-diyl)diformamide **2** as white crystals (1.35 g, yield: 99%, HPLC purity: >95%). ^{1}H NMR (400 MHz, DMSO-d6) δ = 10.53 (d, J = 10.7 Hz, 1H, H_{11}), 10.35 (s, 3H, H_7, H_9), 8.58 (d, J = 10.5 Hz, 1H, H_{12}), 8.48 (d, J = 1.8 Hz, 3H, H_8, H_{10}), 8.08 – 7.91 (m, 7H, H_2, H_3, H_5, H_6), 7.60 – 7.51 (m, 4H, H_1, H_4), 7.47 (t, J = 5.9 Hz, 1H, H_{13}) ppm (Figure S1).

^{13}C NMR (101 MHz, DMSO-d6) δ = 160.5, 133.6, 128.5, 127.9, 125.7, 125.6, 125.4, 124.4, 120.9, 119.3, 118.8 ppm (Figure S2).

HRMS (ESI/Q-TOF) m/z: $[M+H]^+$ Calc. for $C_{12}H_{11}N_2O_2$ 215.0815; found 215.0741.

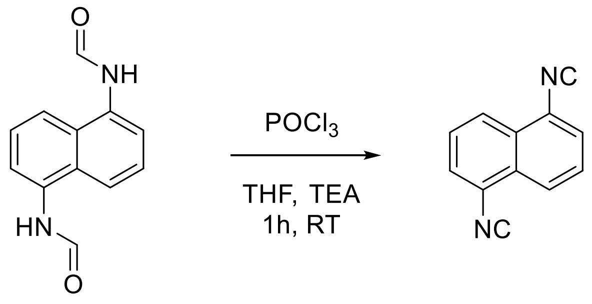

2 **3**

Synthesis of 1,5-diisocyanonaphtalene (3)[B]:

To the mixture of N,N'-(naphthalene-1,5-diyl)diformamide **2** (1.3 g, 6.1 mmol, 1 equivalent), anhydrous tetrahydrofuran (14 ml) and triethylamine (6.77 ml, 48.5 mmol, 8 equivalent), phosphoryl chloride (1.13 ml, 12.1 mmol, 2 equivalent) was added dropwise at 0 °C. The mixture was stirred at room temperature for one hour, followed by HPLC-MS. After the consumption of the formamide, saturated aqueous sodium hydrogen carbonate (16 ml) was added slowly using an ice bath. The mixture was extracted with dichloromethane (3×20 ml) and the combined organic layers were washed with brine (20 ml), then dried over magnesium sulphate. The solvent was removed under reduced pressure, the brownish powder was transferred into a filter, then washed with dimethyl-sulfoxide until its colour has become white, resulting in the desired pure 1,5-diisocyanonaphtalene **3** product (0.67 g, yield: 59%, HPLC purity: 95%). ^{1}H NMR (500 MHz, $CDCl_3$) δ = 8.27 (d, J = 8.4 Hz, 2H, H_3, H_6), 7.73 (d, J = 7.4 Hz, 2H, H_1, H_4), 7.67 (t, J = 7.9 Hz, 2H, H_2, H_5) ppm (Figure S3).

^{13}C NMR (126 MHz, $CDCl_3$) δ = 169.5, 128.5, 127.4, 126.2, 124.9 ppm (Figure S4).

HRMS (ESI/Q-TOF) m/z: $[M+H]^+$ Calc. for $C_{12}H_6N_2$ 179.0609; found 179.0588.

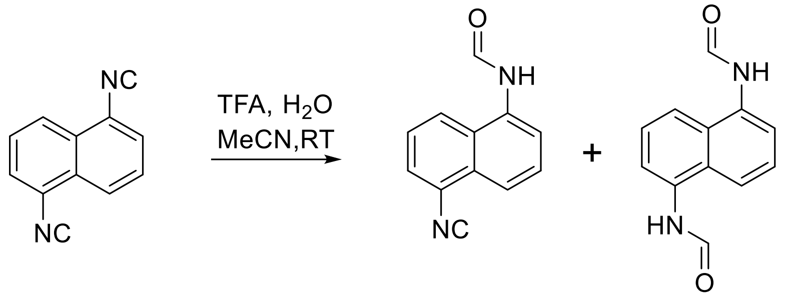

3 **4** **2**

Synthesis of N-(5-isocyanonaphthalen-1-yl)formamide (4):

To the mixture of 1,5-diisocyanonaphtalene **3** (150 mg, 0.84 mmol, 1 equivalent), acetonitrile (3 ml) and distilled water (0.15 ml, 10 equivalent), trifluoroacetic acid (120 µl, 1.57 mmol, 1.9 equivalents) was added dropwise at 0 °C in 80 minutes. The ratio of the mono- (**4**) and diformamide (**2**) products was monitored by HPLC-MS. The product **4** was isolated by preparative HPLC (Gradient elution, 15%→45% acetonitrile in aqueous ammonium-hydrogen-carbonate solution(0.4 g in 1 L water)). N-(5-isocyanonaphthalen-1-yl)formamide **4**, was isolated as white powder (29 mg, yield: 17%, HPLC purity: >95%). ^{1}H NMR (400 MHz, DMSO-d6) δ = 10.68 (s, 1H, H_9), 10.51 (s, 1H, H_7), 8.64 (d, *J* = 5.6 Hz, 1H, H_{10}), 8.51 (d, *J* = 1.6 Hz, 1H, H_8), 8.30 (dd, *J* = 8.8, 3.9 Hz, 2H, H_4), 8.16 (d, *J* = 7.6 Hz, 1H, H_3), 7.92 (dd, *J* = 12.9, 8.1 Hz, 4H, H_1, H_6), 7.80 – 7.69 (m, 2H, H_2), 7.69 – 7.59 (m, 3H, H_5, H_{11}) ppm (Figure S5).
^{13}C NMR (101 MHz, DMSO-d6) δ = 160.5, 133.6, 128.5, 127.9, 125.7, 125.6, 125.4, 124.4, 120.9, 119.3, 118.8 ppm (Figure S6).

HRMS (ESI/Q-TOF) m/z: $[M+H]^+$ Calc. for $C_{12}H_9N_2O$ 197.0709; found 197.0652.

[A]https://doi.org/10.1016/j.bmcl.2014.05.071

[B]https://doi.org/10.1002/open.202000250

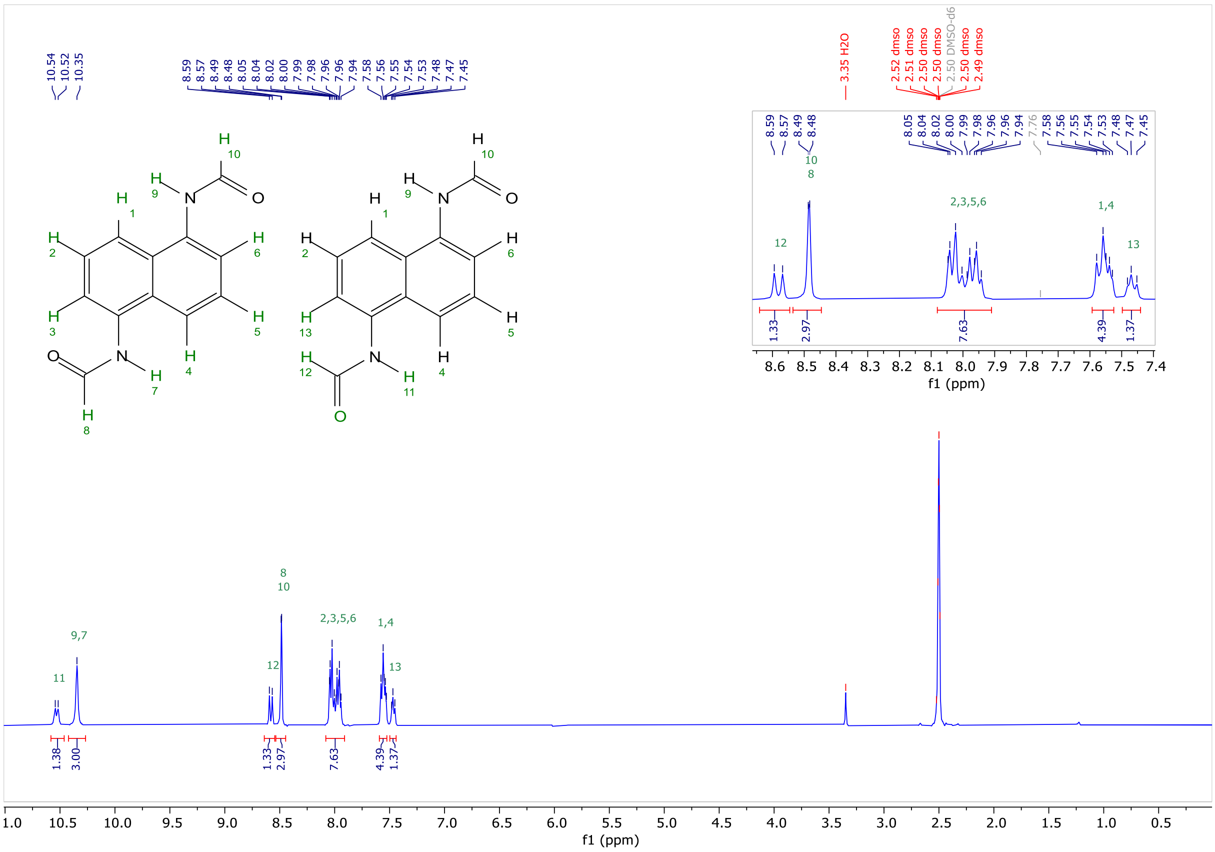

Figure S1. ^{1}H NMR spectrum of compound **2** recorded at 400 MHz in DMSO-d6.

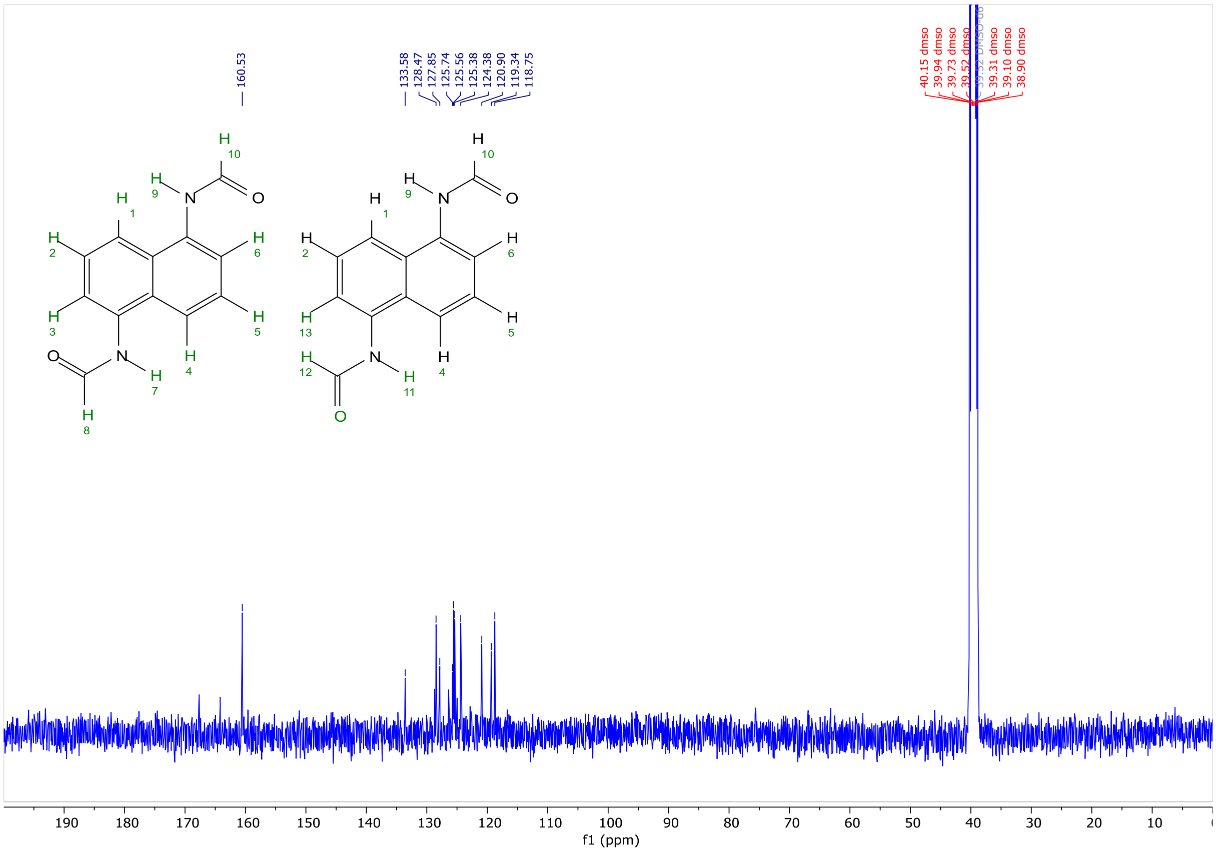

Figure S2. ^{13}C NMR spectrum of compound **2** recorded at 101 MHz in DMSO-d6.

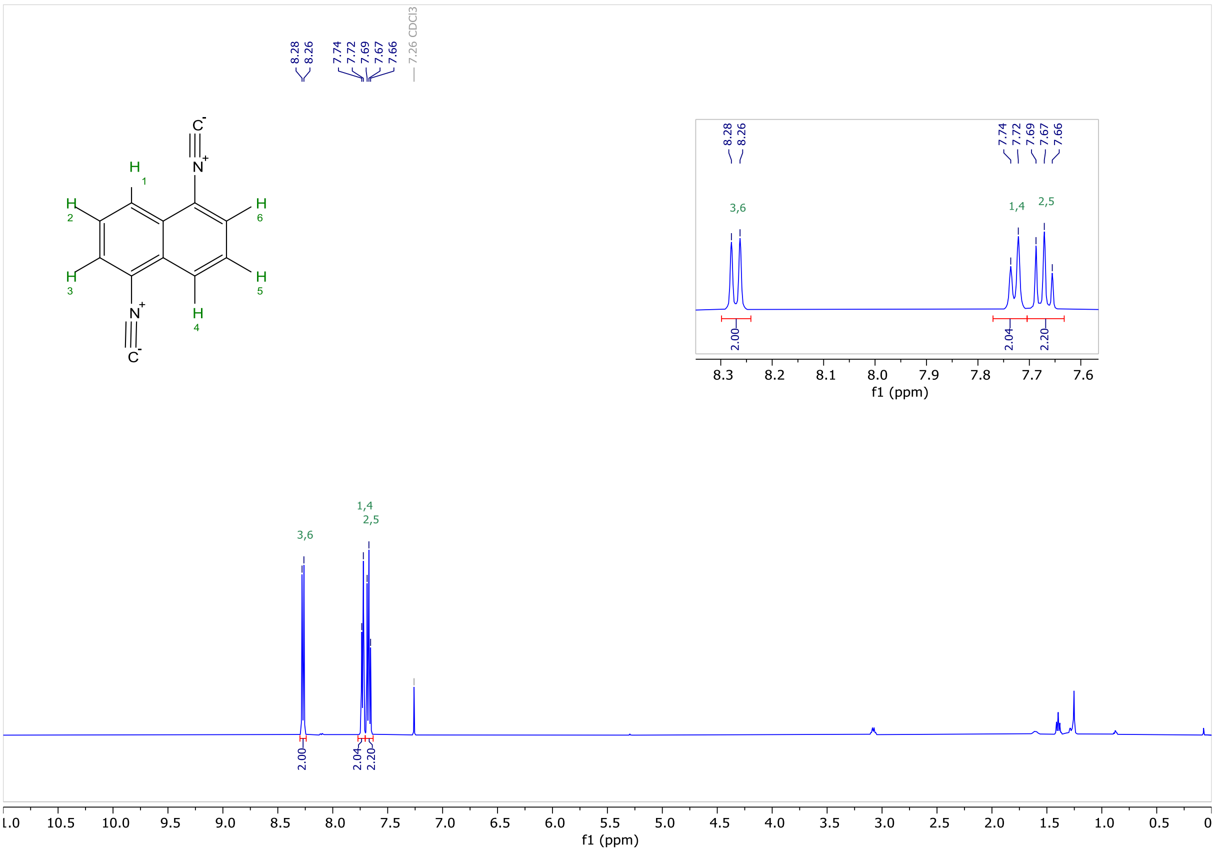

Figure S3. ^{1}H NMR spectrum of compound **3** recorded at 500 MHz in $CDCl_3$.

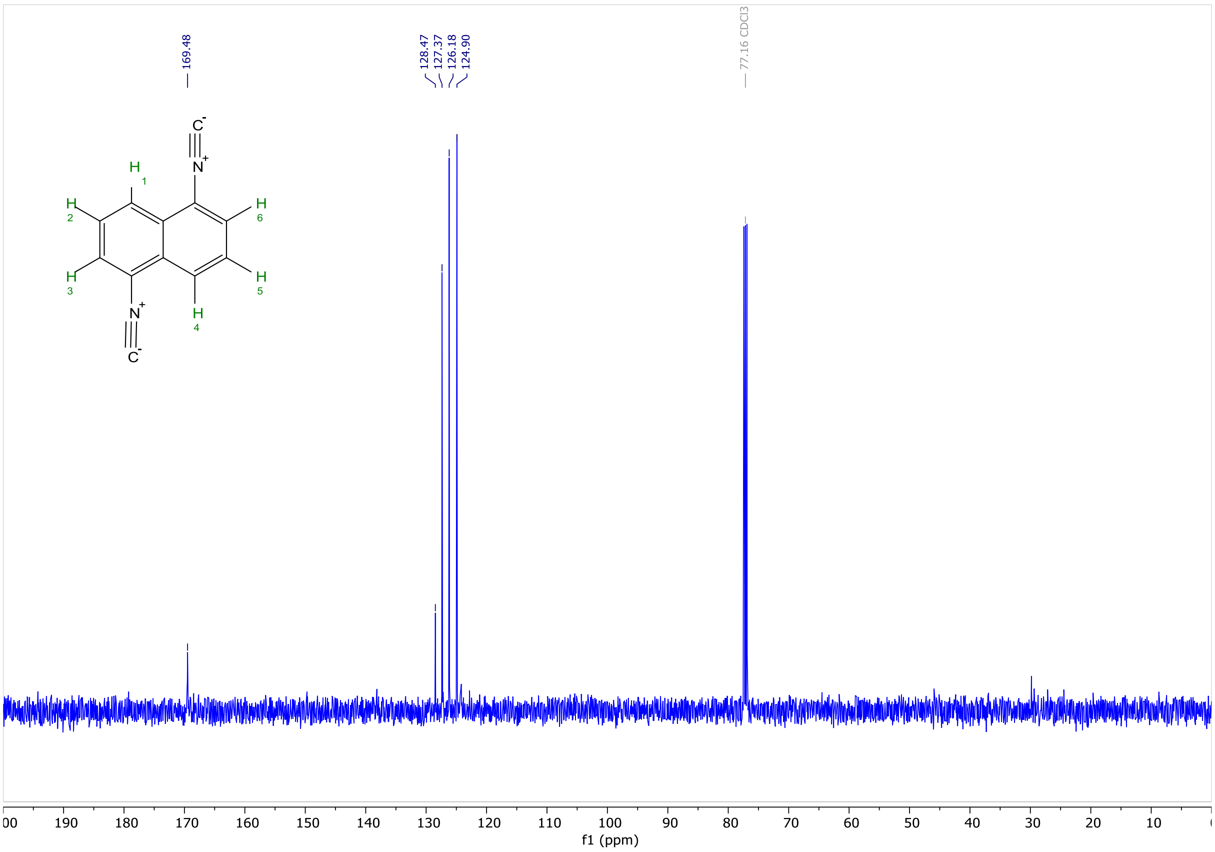

Figure S4. ^{13}C NMR spectrum of compound **3** recorded at 126 MHz in $CDCl_3$.

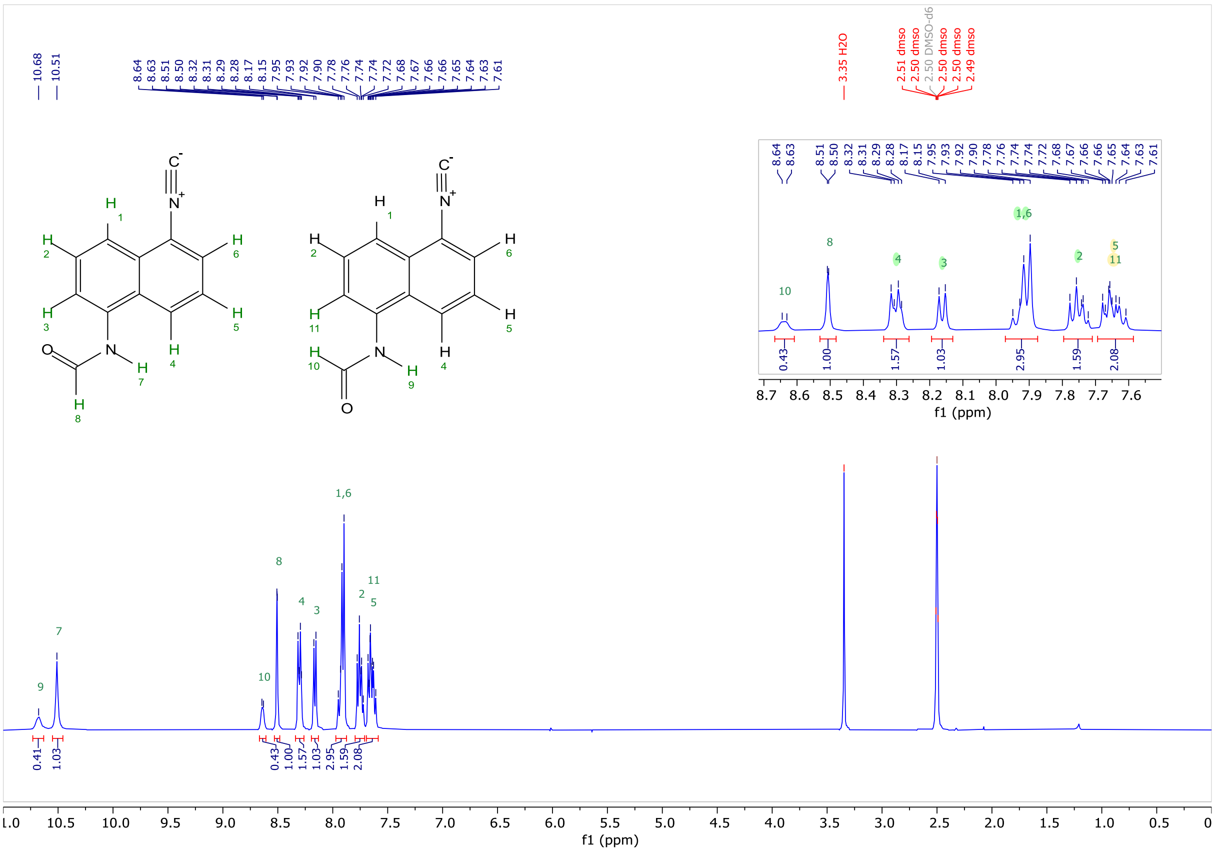

Figure S5. ^{1}H NMR spectrum of compound **4** recorded at 400 MHz in DMSO-d6.

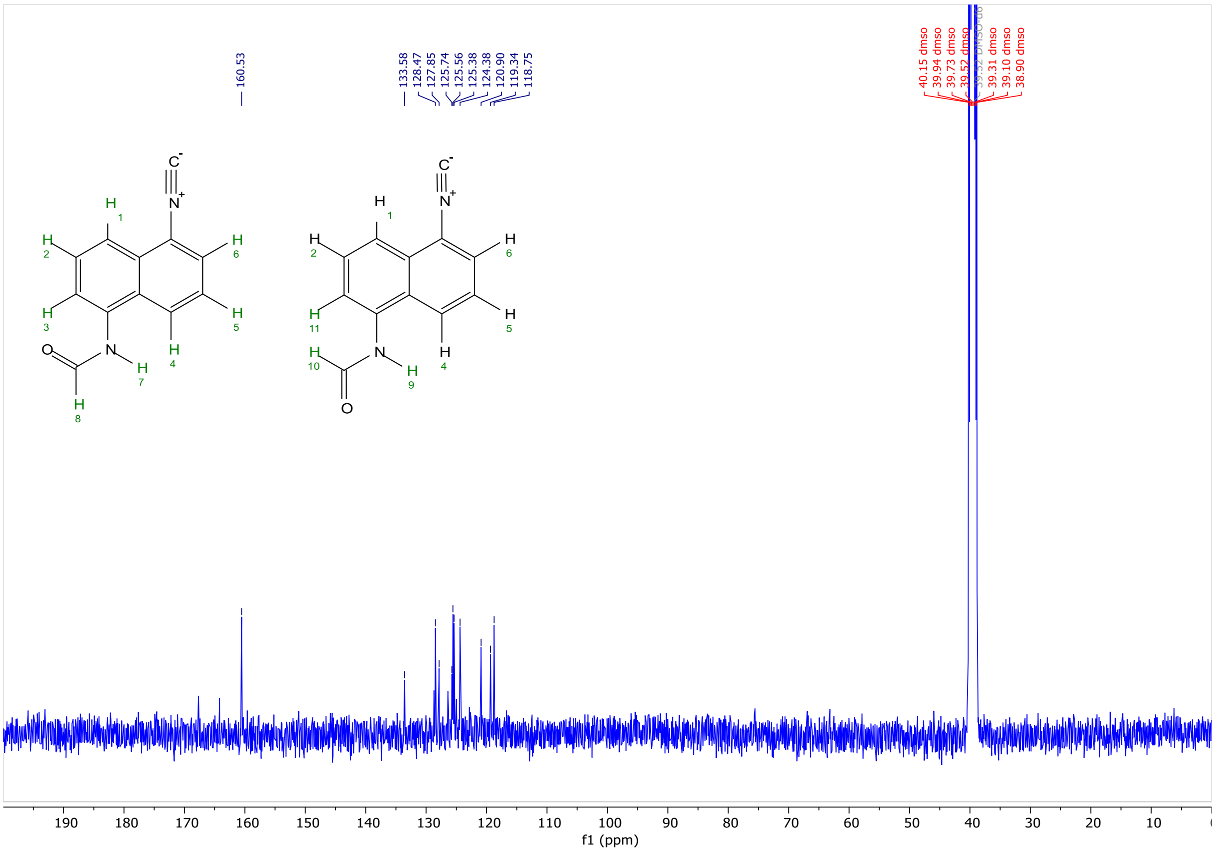

Figure S6. ^{13}C NMR spectrum of compound **4** recorded at 101 MHz in DMSO-d6.

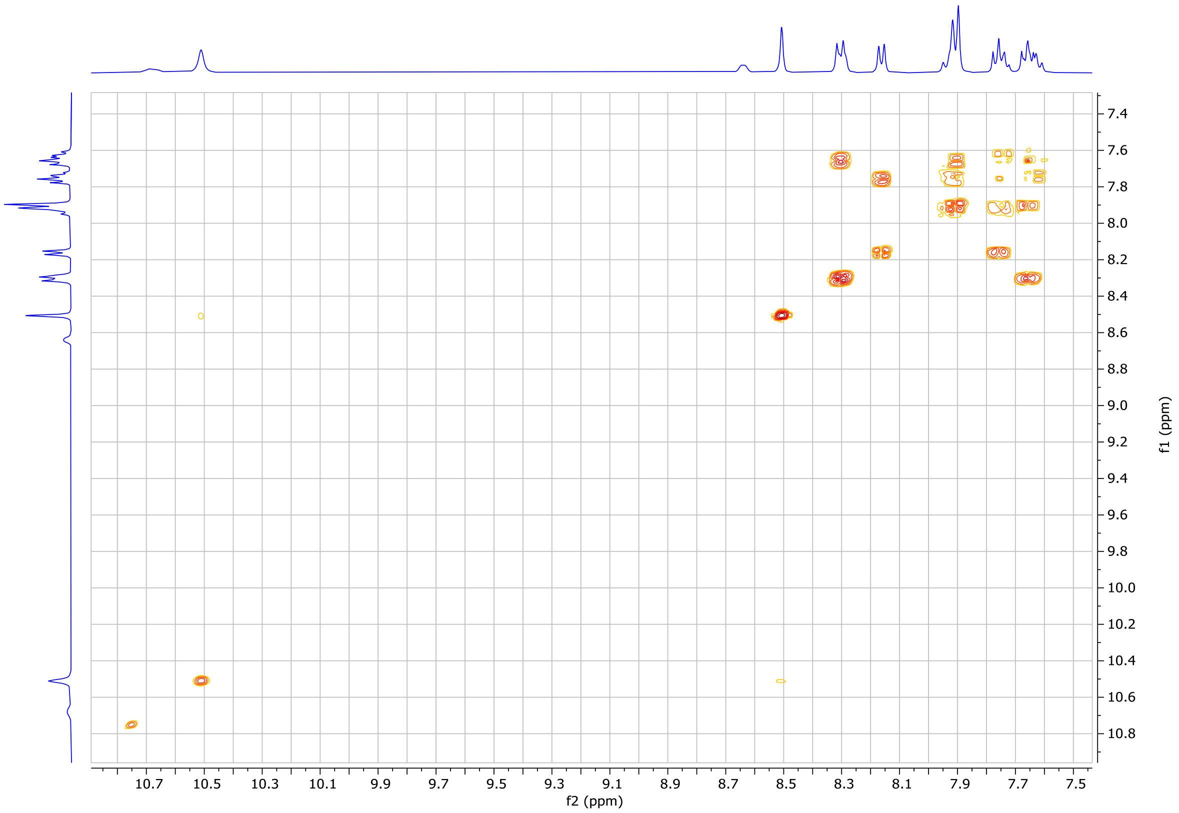

Figure S7. COSY (Correlated Spectroscopy) spectrum of compound **4** recorded at 400 MHz in DMSO-d6.

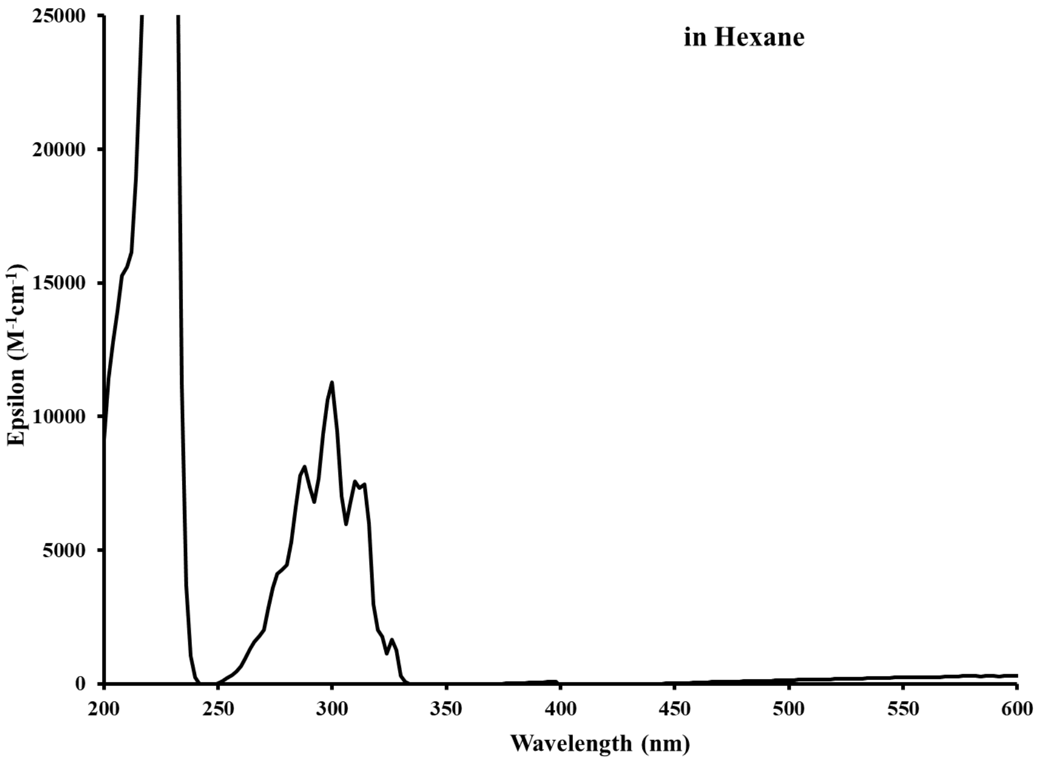

Figure S8. UV-Vis absorbance spectrum of DIN recorded in hexane.

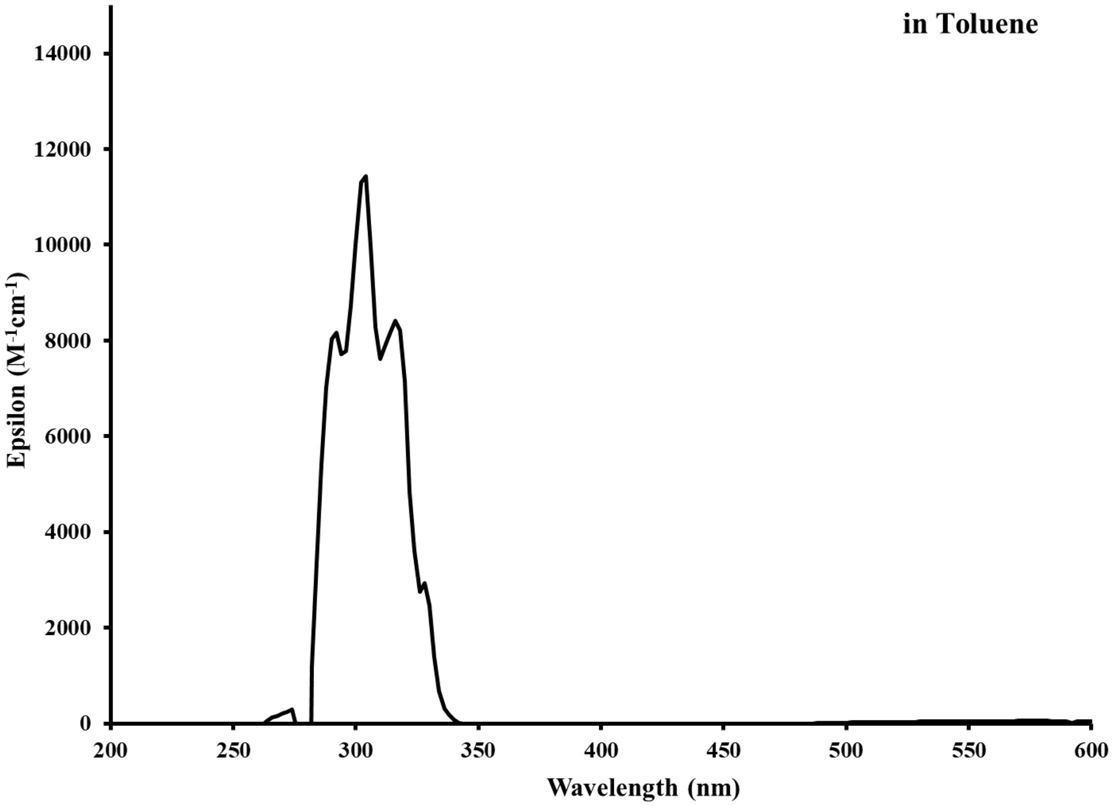

Figure S9. UV-Vis absorbance spectrum of DIN recorded in toluene.

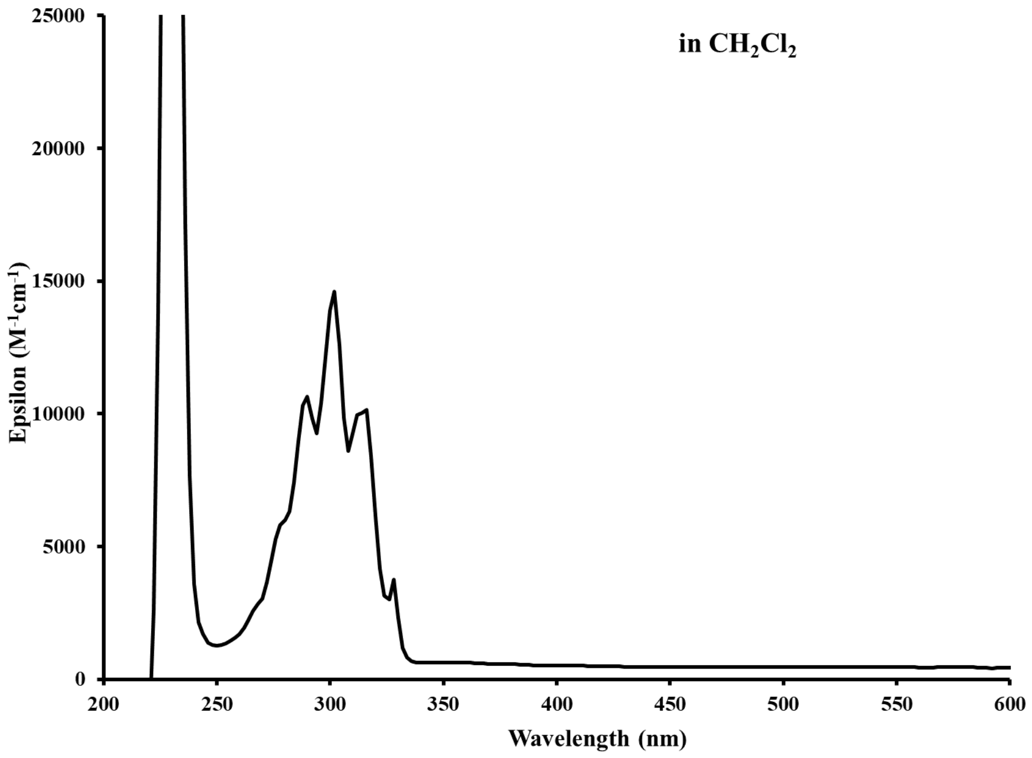

Figure S10. UV-Vis absorbance spectrum of DIN recorded in methylene-chloride.

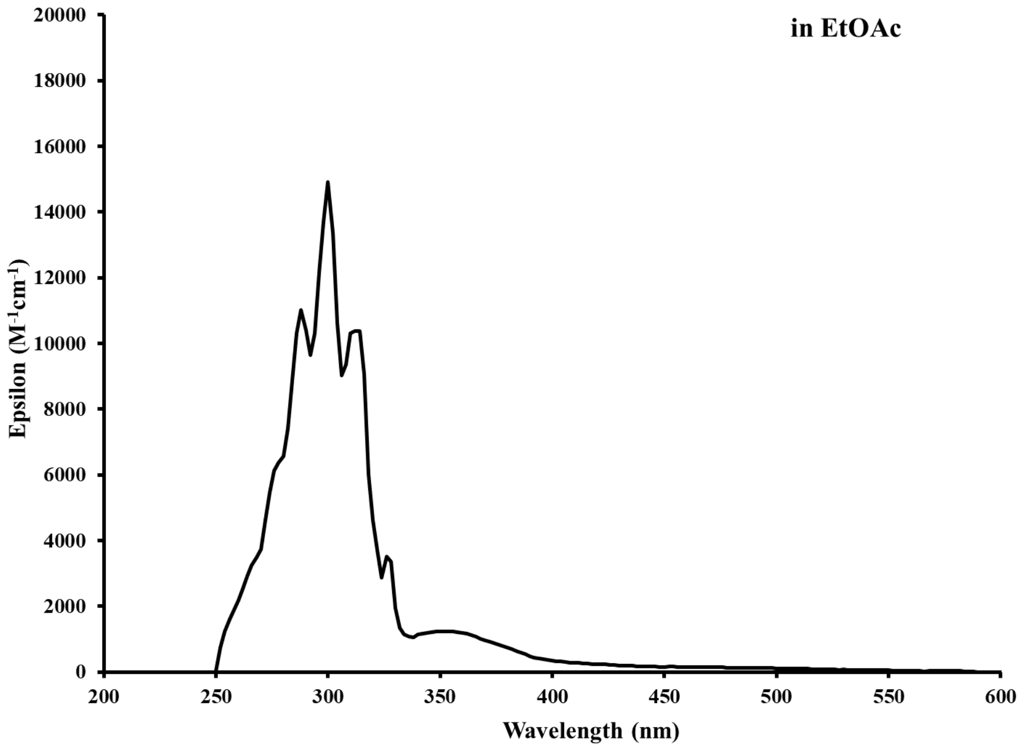

Figure S11. UV-Vis absorbance spectrum of DIN recorded in ethyl acetate.

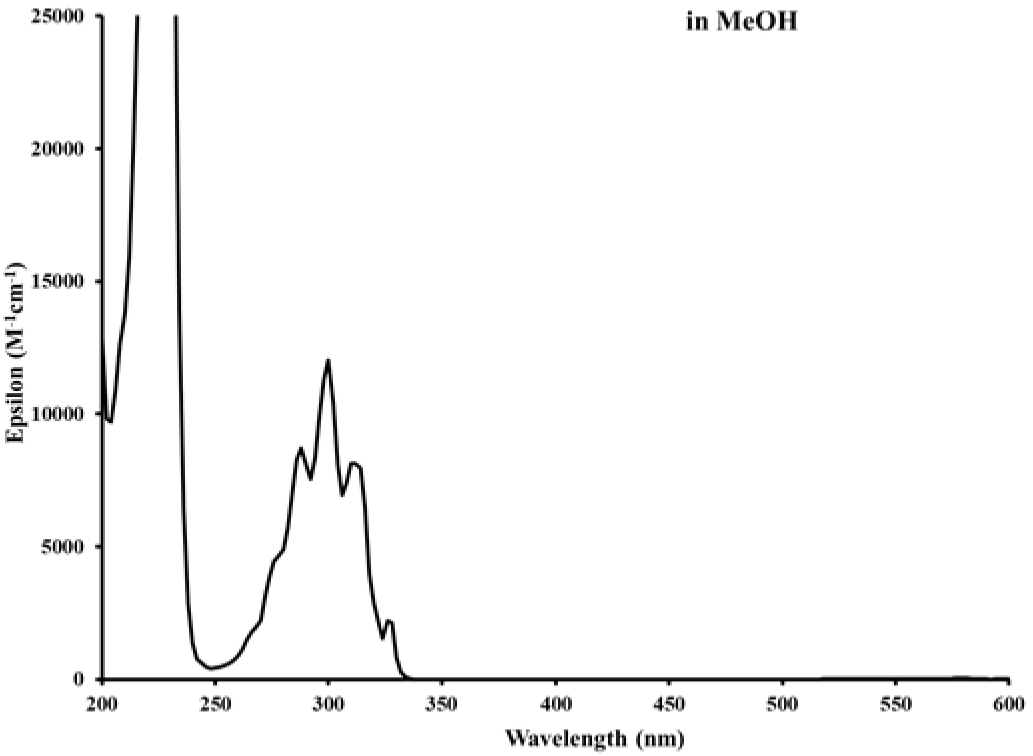

Figure S12. UV-Vis absorbance spectrum of DIN recorded in methanol.

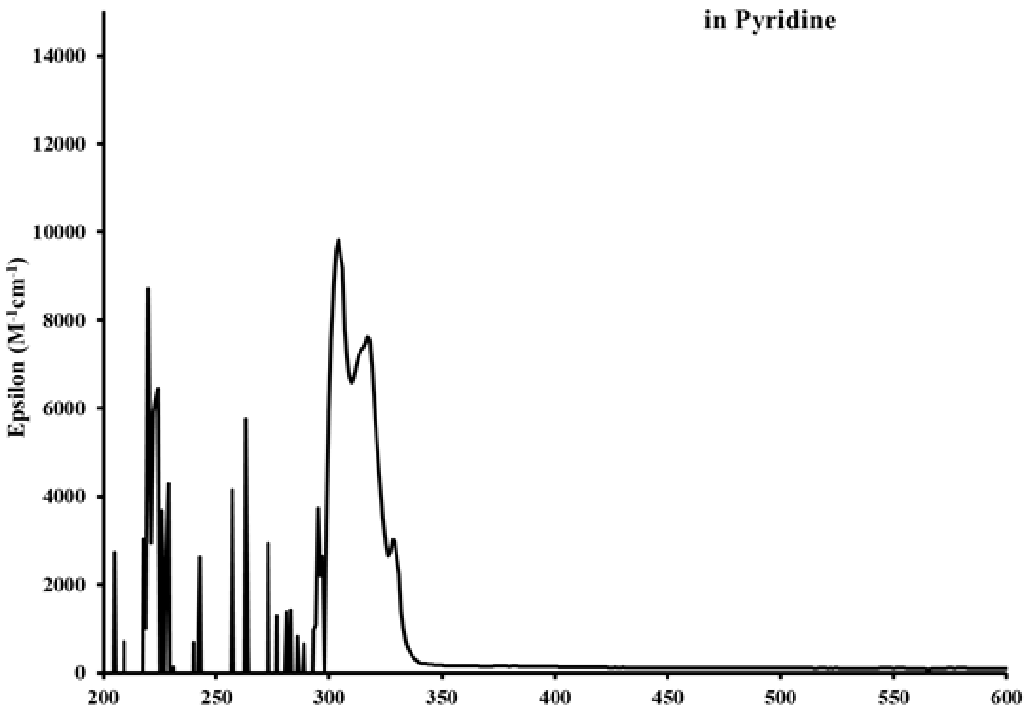

Figure S13. UV-Vis absorbance spectrum of DIN recorded in pyridine.

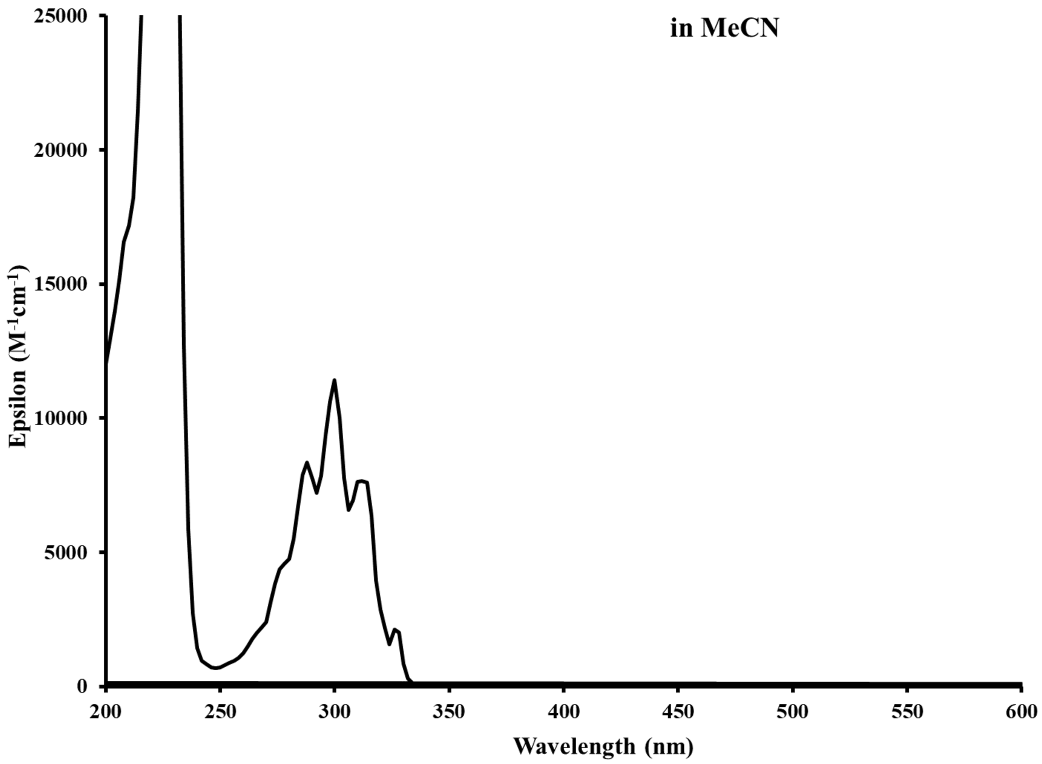

Figure S14. UV-Vis absorbance spectrum of DIN recorded in acetonitrile.

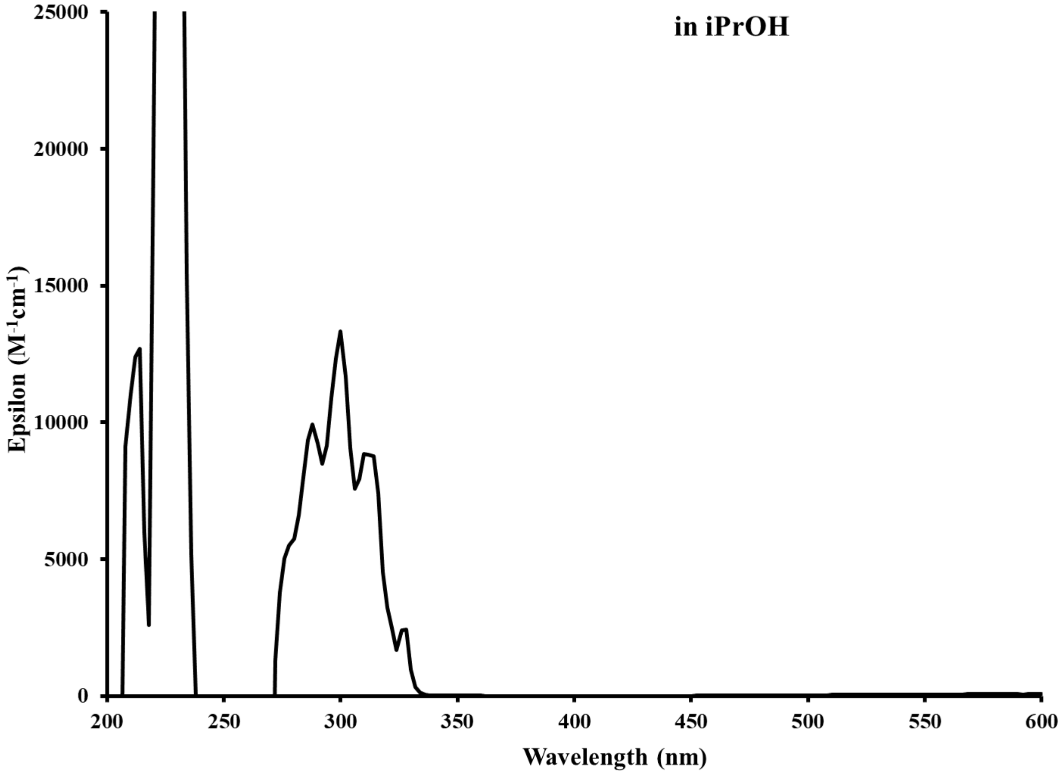

Figure S15. UV-Vis absorbance spectrum of DIN recorded in 2-propanol.

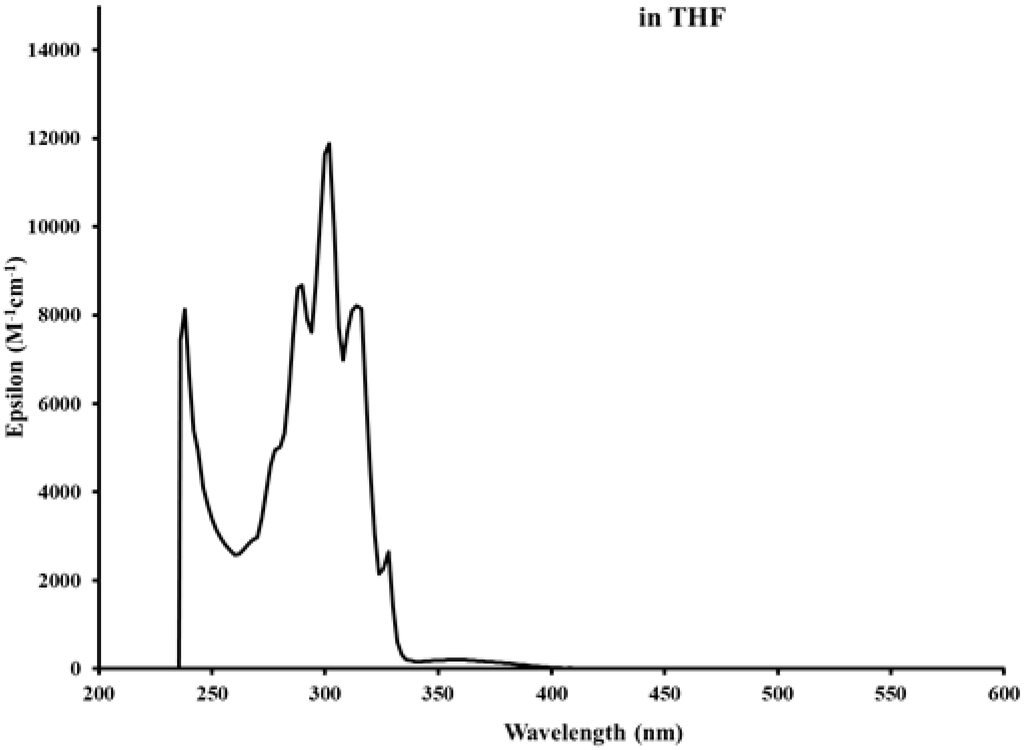

Figure S16. UV-Vis absorbance spectrum of DIN recorded in tetrahydrofuran.

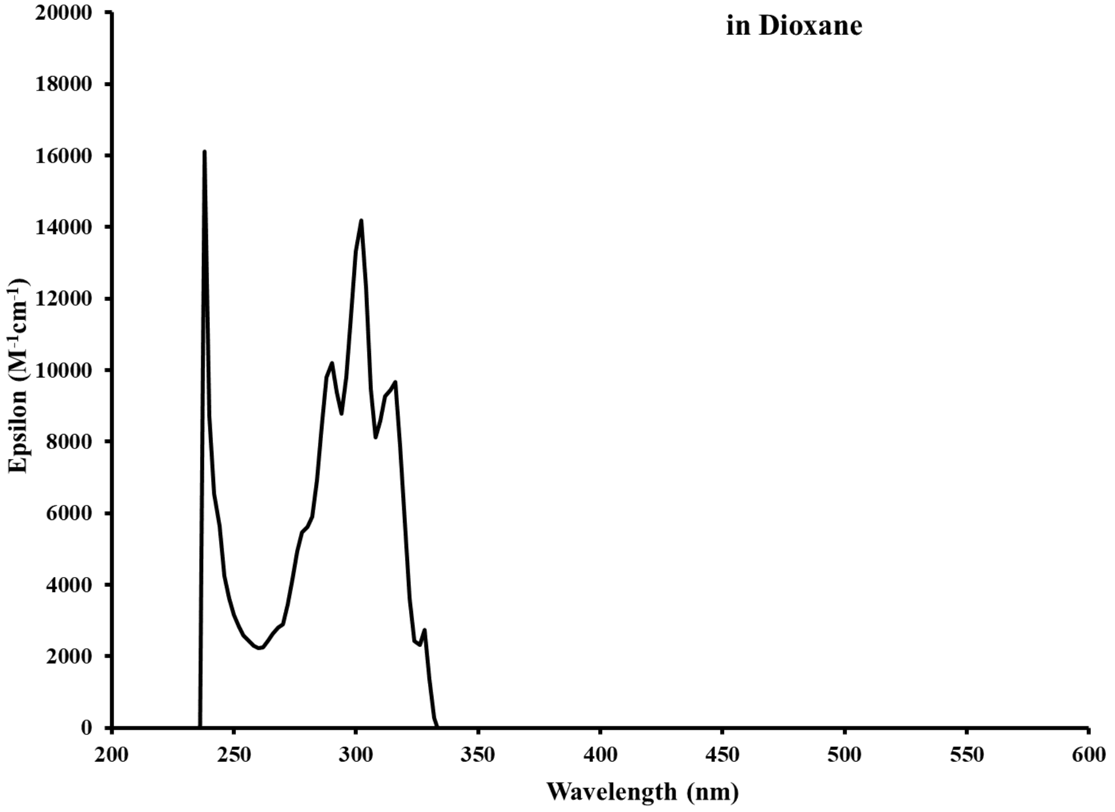

Figure S17. UV-Vis absorbance spectrum of DIN recorded in dioxane.

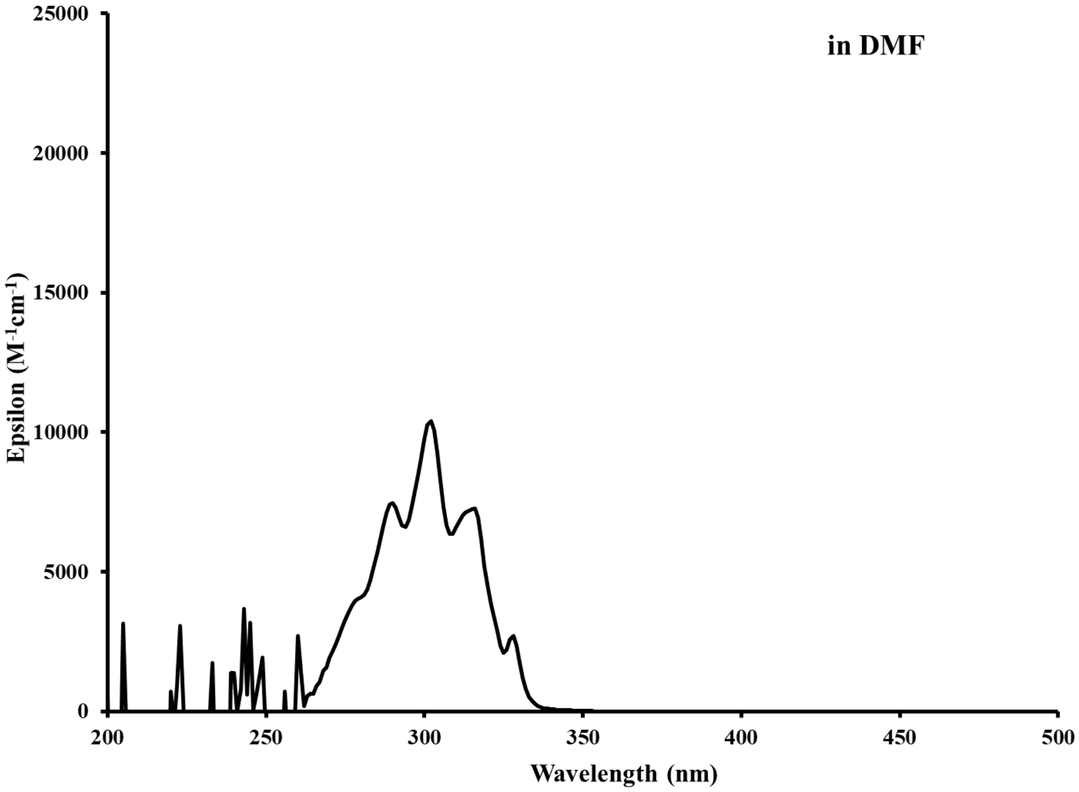

Figure S18. UV-Vis absorbance spectrum of DIN recorded in dimethylformamide.

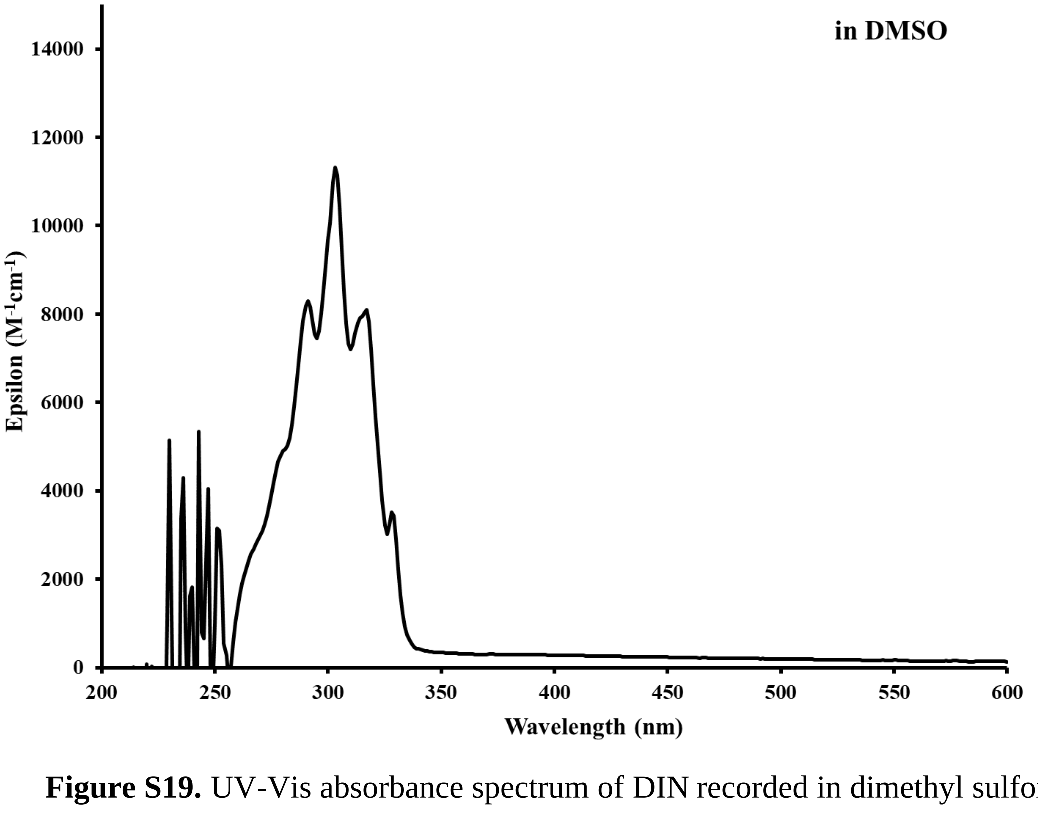

Figure S19. UV-Vis absorbance spectrum of DIN recorded in dimethyl sulfoxide.

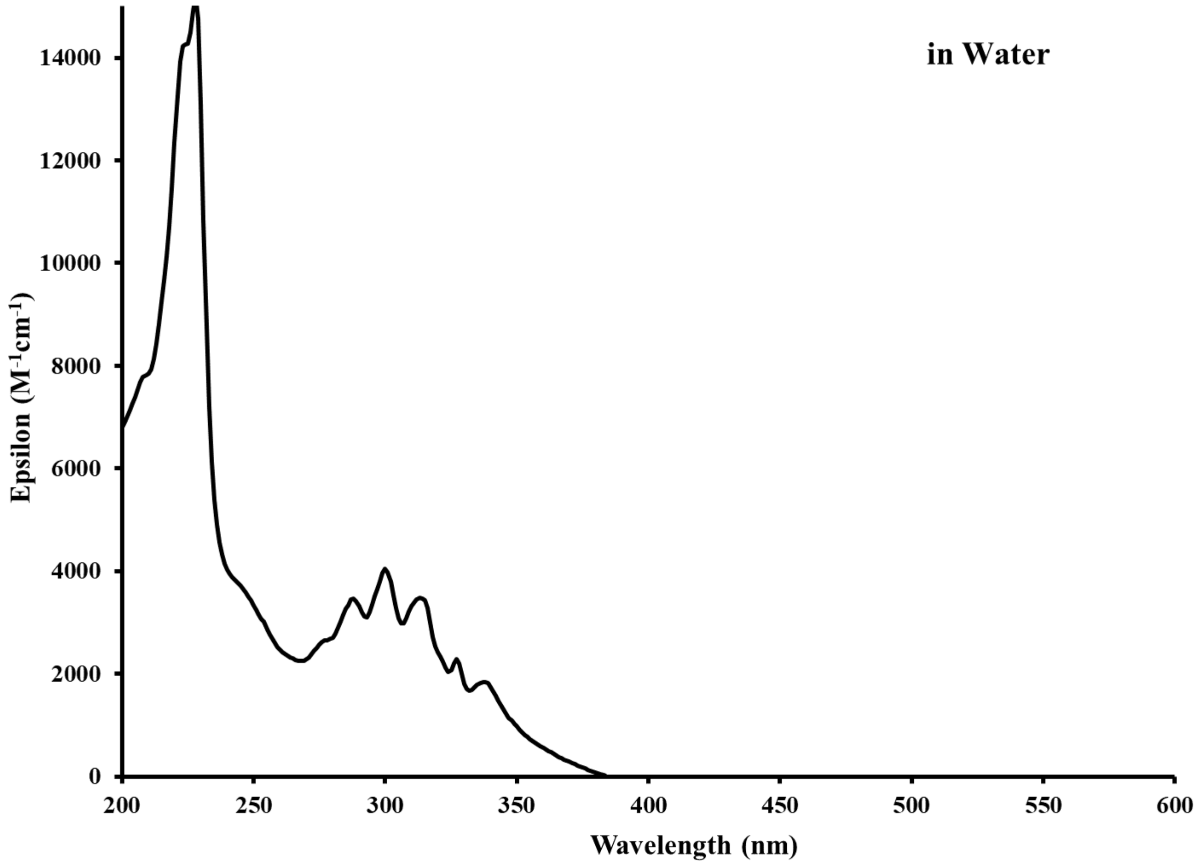

Figure S20. UV-Vis absorbance spectrum of DIN recorded in water.
